# PathBIX—a web server for network-based pathway annotation with adaptive null models

**DOI:** 10.1093/bioadv/vbab010

**Published:** 2021-07-01

**Authors:** Miguel Castresana-Aguirre, Emma Persson, Erik L L Sonnhammer

**Affiliations:** Department of Biochemistry and Biophysics, Science for Life Laboratory, Stockholm University, Stockholm 17121, Sweden

## Abstract

**Motivation:**

Pathway annotation is a vital tool for interpreting and giving meaning to experimental data in life sciences. Numerous tools exist for this task, where the most recent generation of pathway enrichment analysis tools, network-based methods, utilize biological networks to gain a richer source of information as a basis of the analysis than merely the gene content. Network-based methods use the network crosstalk between the query gene set and the genes in known pathways, and compare this to a null model of random expectation.

**Results:**

We developed PathBIX, a novel web application for network-based pathway analysis, based on the recently published ANUBIX algorithm which has been shown to be more accurate than previous network-based methods. The PathBIX website performs pathway annotation for 21 species, and utilizes prefetched and preprocessed network data from FunCoup 5.0 networks and pathway data from three databases: KEGG, Reactome, and WikiPathways.

**Availability:**

https://pathbix.sbc.su.se/

**Contact:**

erik.sonnhammer@scilifelab.se

**Supplementary information:**

Supplementary data are available at *Bioinformatics Advances* online.

## 1. Introduction

Data generation is often not the limiting factor to gain biological insight. High-throughput experiments provide a plethora of data, which in turn yields a list of genes or proteins that are related to the studied phenotype. However, this list alone is not enough to understand the complex biological mechanisms that are involved in a given condition. An indispensable approach to resolve this issue is pathway enrichment analysis, which can reveal activated biological processes by detecting patterns of gene groups in the list. This is done by comparing to functionally characterized and curated gene sets, known as pathways, in databases such as KEGG (Kanehisa *et al.*, 2021), Reactome (Fabregat *et al.*, 2018), WikiPathways (Martens *et al.*, 2021), NCIPathways (Schaefer *et al.*, 2009) and Pathway Commons (Rodchenkov *et al.*, 2020).

The importance of pathway enrichment analysis can be appreciated from the large number of methods available (Khatri *et al.*, 2012; Nguyen *et al.*, 2019). Pathway enrichment methods can be divided into two main approaches: methods that use a score for every gene in the genome, and methods that only use a set of relevant genes. If gene expression data are available, the former approach is reasonable. However, experimental data often do not offer genome-wide per-gene scores, for instance from Genome-Wide Association Studies (GWAS), protein–protein interactions, curated gene-disease associations or gene–drug associations . For such cases, relevant gene set methods are more suitable since they do not require scoring every gene. Among such methods, the most widely used web server, DAVID (Huang *et al.*, 2009), relies on the gene overlap between the gene set and the pathway. Pathway enrichment is assessed by comparing the observed overlap with the overlap that is expected by chance with a modified Fisher’s exact test called the EASE score. Despite being the most widely used approach, overlap-based methods have some important limitations. They assume gene independence which is often not true in biology since genes interact between each other in complex biological systems. Moreover, they do not take into account that some genes in the gene set may have a more important role in the pathway than others. Additionally, these methods are highly dependent on the coverage of pathway knowledge, which is still incomplete, leading to false negatives (Castresana-Aguirre and Sonnhammer, 2020; Ogris *et al.*, 2017). For instance, if half of a pathway is used as a query against the other half, it would not be possible to detect enrichment.

Sensitivity can be substantially boosted by network-based approaches that do not rely on the overlap between query set and pathway but instead measure the enrichment of network crosstalk between them. To this end, they employ a functional association network of genes such as FunCoup (Persson *et al.*, 2021) or STRING (Szklarczyk *et al.*, 2021). By using interactions between query gene set and pathway instead of the gene overlap, the pathway incompleteness problem is diminished. Some of these methods are implemented in web servers, for example, PathwAX (Ogris *et al.*, 2016), EviNet (Jeggari *et al.*, 2018) and Enrichnet (Glaab *et al.*, 2012). However, these web servers have some limitations. Enrichnet does not estimate the statistical significance of the crosstalk, and PathwAX and EviNet sometimes suffer from high false-positive rates, which can be observed when using random query gene sets (Castresana-Aguirre and Sonnhammer, 2020). The reason for false positives is that the null model of these methods assumes that pathways follow the statistical properties of random gene sets, which is not true since pathways tend to contain functionally related genes. This issue has been addressed by the novel method ANUBIX (Castresana-Aguirre and Sonnhammer, 2020), which scores random gene sets against the real pathways to build a custom null model for each pathway. By keeping pathway properties intact it can model random crosstalk much better than previous methods, thereby decreasing the false-positive rate while keeping a high true-positive rate.

To make ANUBIX more accessible to the scientific community, we developed a web server named PathBIX, featuring a graphical web interface with preprocessed data in front of an ANUBIX back-end. It supports 21 species and three pathway databases: KEGG, Reactome and WikiPathways. To visualize the results, the connectivity of each query gene to each pathway is shown as a heatmap matrix, and an interactive bipartite network is provided for each query-pathway pair.

## 2. Methods

### 2.1 Implementation of web application

The PathBIX application is built with a frontend application containing the graphical interface, using the Angular (v.9) framework (https://angular.io) , and a backend application containing the application logic, built as a Python (v.3.7) (Van Rossum and Drake, 2009) rest API. The Python API runs the R (v.3.6) (R Core Team, 2017) version of the ANUBIX (Castresana-Aguirre and Sonnhammer, 2020) algorithm to perform the pathway enrichment analysis. Only genes present in the FunCoup networks are used as background to sample genes for the null model. The Python API uses precalculated data stored in a Postgres (v.10) (Stonebraker and Rowe, 1986) database. The database and application are deployed and run in Podman (v.2.0.5) (https://podman.io) containers, and the containers are run on a CentOS8 virtual machine.

### 2.2 Networks and species

The networks used to calculate the crosstalk between the query and the pathway gene sets were collected from FunCoup 5.0 (Persson *et al.*, 2021), a comprehensive database of functional association networks built on redundancy-weighted naive Bayesian integration of 11 evidence types. PathBIX uses all 21 species available in the latest FunCoup release. In PathBIX, the networks are used with a cutoff on the link confidence in order to ensure high reliability on the links. The default link confidence cutoff used for the networks is 0.8, but the user may select even higher thresholds of 0.95 or 0.99. The number of links and genes included in the networks for each PathBIX species, using the default cutoff of 0.8 can be found in Supplementary Table S1.

Gene identifier mappings were extracted from the FunCoup 5.0 database and incorporated into PathBIX, allowing the user to input and label their results as either Ensembl Gene IDs, Ensembl Protein IDs, Ensembl Transcript IDs, Gene symbols, UniProt ID, NCBI gene ID or a selection of species-specific database identifiers, depending on the available identifier types for each gene. These mappings originate from idmapping files from UniProt (v.2020_01) (UniProt Consortium, 2021) and the EBI Quest for Orthologs reference proteomes (v.2020_04) (Altenhoff *et al.*, 2020). The networks and mapping data retrieved from FunCoup will be updated in the PathBix resource when a new version of the FunCoup database is available.

### 2.3 Pathway databases

PathBIX uses information on pathway gene sets from three different pathway databases, KEGG (Kanehisa *et al.*, 2021) (v.96.0), Reactome (v.75) (Fabregat *et al.*, 2018) and WikiPathways (Martens *et al.*, 2021) (v.20201110). These databases were chosen as they are highly cited and complementary open-source pathway databases (Mubeen *et al.*, 2020). The sets of pathway genes, together with the pathway identifier, pathway name and pathway class were fetched from the respective pathway database website and processed so that all gene identifiers were translated to Ensembl IDs. For WikiPathways, data included in the set of ‘reviewed and approved’ pathways were processed and translated, and species resulting in having more than five pathways were included in the PathBIX pathway collection. Pathways having a WikiPathway category were assigned this category name as their pathway class. Reactome pathways are hierarchically distributed, with the lowest level pathways being very specific. Once mapped to FunCoup, 46% of the pathway leaf nodes have less than 10 genes. To avoid very small pathways and reduce the number of pathways, we flattened Reactome’s hierarchy by merging child pathways with their parents until a pathway size distribution similar to KEGG was obtained for each species. We intend to perform a yearly update to the PathBIX database to ensure that the pathway data used in the analysis is recent and up to date.

### 2.4 Examples

For each species in PathBIX, at least one example gene set is provided on the website, in order to make it easy for users to see what the expected input looks like, and to test the functionality of the website. The examples were fetched from MSigDB (Subramanian *et al.*, 2005) for *Homo sapiens* and *Mus musculus*, Gramene ontology (Jaiswal *et al.*, 2002) for *Arabidopsis thaliana*, PomBase (Lock *et al.*, 2020) for *Schizosaccharomyces pombe* and Gene Ontology (Carbon *et al.*, 2009) for the remaining species. Examples were selected so that a sufficient amount of genes could be mapped and identified in the networks to give reliable and relevant results.

## 3. Results

### 3.1 The PathBIX web server

**Fig. 1.**
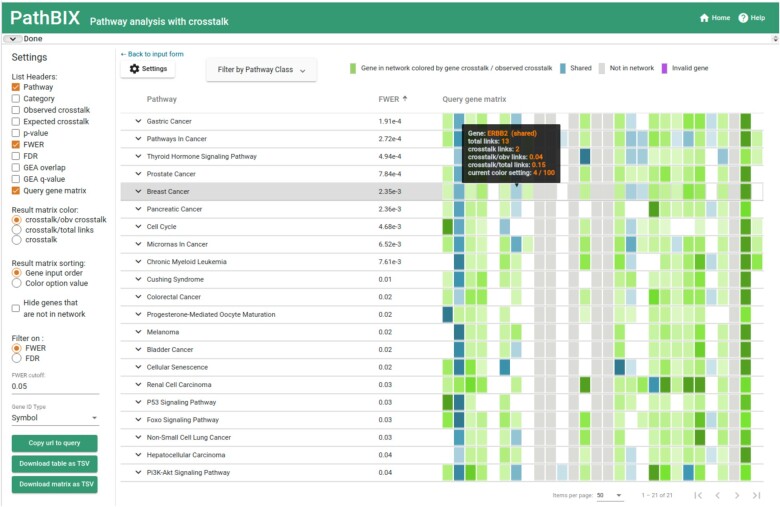
The PathBIX result page when running the example gene set CHIN_BREAST_CANCER_COPY_NUMBER_UP for *H.sapiens* against the KEGG pathway database with a network cutoff of 0.95. The resulting enriched pathways can be seen in the table in the middle together with their FWER values. The left panel contains settings that control what is shown in the table, for example that FWER must be lower than 0.05. On the right, the colored rectangular areas form a matrix that shows the crosstalk of each query gene against each found pathway, where the intensity of the color increases with higher crosstalk. Each column in the matrix represents a query gene, and information about the gene’s ID and its crosstalk to the pathway in question is shown in a mouseover box (black background). Green areas indicate genes with crosstalk to the pathway, and blue rectangles represent query genes shared with the pathway

**Fig. 2.**
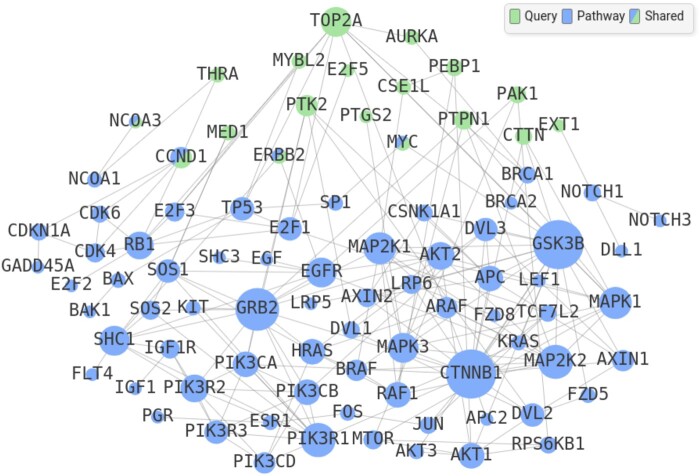
Detail of the PathBIX results page. The network viewer showing the crosstalk between pathway number 5 in Figure 1, ‘Breast cancer’ and the query gene set CHIN_BREAST_CANCER_COPY_NUMBER_UP. Nodes marked in green represent query genes while nodes marked in blue represent pathway genes. Nodes colored green and blue are genes that overlap between the query and the pathway gene set. The network viewer is toggled on or off by clicking on any pathway in the results table

The PathBIX website is an openly available online resource to perform reliable network-based pathway enrichment analysis. The website requires the user to input a query gene set, select one of 21 available species, one of three available pathway databases, and a network confidence cutoff for the crosstalk analysis. The results of the analysis are displayed as a table of enriched pathways as well as an interactive network visualization of the crosstalk between the query gene set and the pathway genes (see Fig. 1). The pathway table by default displays pathways having a family-wise error rate (FWER) lower than 0.05 to ensure high reliability of the listed pathways. This cutoff can be adapted to show pathways having a higher or lower FWER, or to be filtered by false discovery rate (FDR). The network view can be expanded for each of the pathways listed and allows the user to move nodes, as well as to hover over nodes in order to see additional gene information, including gene identifiers and description (see Fig. 2).

To give the user an overview of the crosstalk between the query and the pathways, the pathway table contains a matrix showing all query genes, colored by the level of crosstalk to the pathway genes in the respective pathway. The way that the coloring is displayed can be modified by the user to show either genes colored by gene crosstalk over the observed crosstalk, gene crosstalk over the total links per gene, or absolute crosstalk. The results view is highly customizable, and lets the user adapt the visible results in accordance to its needs, by for example displaying pathway category, observed and expected crosstalk, *P*-value, FDR and FWER as well as comparisons of the PathBIX results to standard Gene Enrichment Analysis (GEA). All results can be downloaded as text files or network images in various formats (.svg, .png, .pdf) to be used for further analysis. The results of PathBIX can easily be stored or shared by using the shareable URL, which will allow the users to quickly return to their results.

### 3.2 Data in PathBIX

**Fig. 3.**
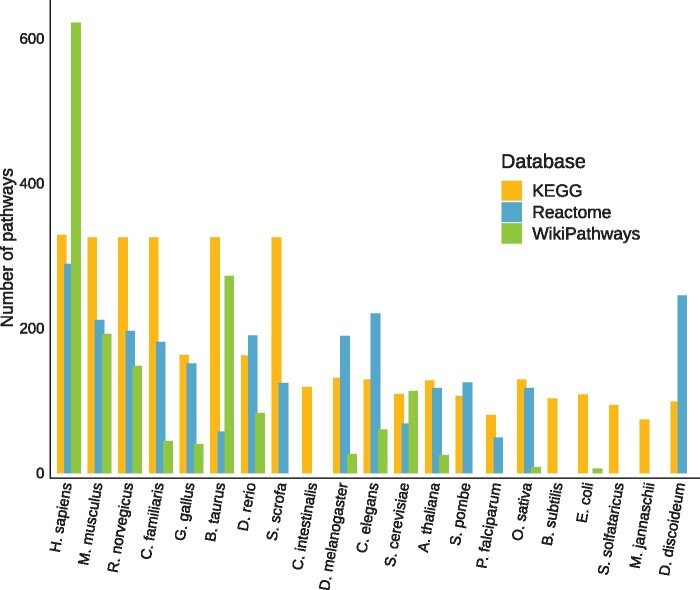
Number of pathways included in PathBIX, from KEGG, Reactome and WikiPathways, for each species in PathBIX

**Fig. 4.**
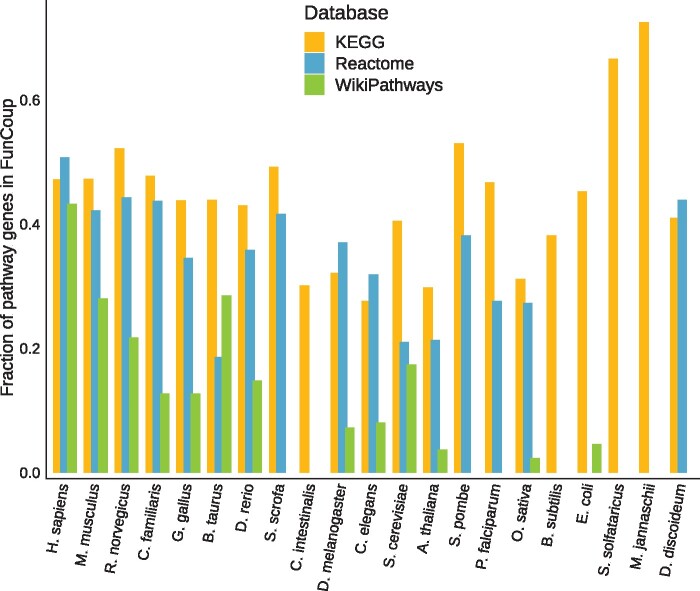
Fraction of genes in the FunCoup 5 networks with a confidence cutoff of 0.8 that are represented among the unique genes retrieved from the respective pathway database, for all species in PathBIX

The integration of data from the three pathway databases, KEGG, Reactome and WikiPathways into PathBIX resulted in a total of 7856 pathways in the PathBIX database. The number of pathways included in PathBIX for each of the pathway databases, for each species, can be seen in Figure 3. The number of pathways ranges between 329 pathways for *H.sapiens* and 74 pathways for *Methanocaldococcus jannaschii* in KEGG, 289 pathways for *H.sapiens* and 49 pathways for *Plasmodium falciparum* in Reactome, and 622 pathways for *H.sapiens* and 6 pathways for *Escherichia coli* in WikiPathways. For Reactome and WikiPathways, pathway information is missing for some of the species present in PathBIX, while KEGG pathways are available for all species. The number of unique pathway genes included in the PathBIX database varies between the pathway databases, and since ANUBIX is basing the analysis on the information in the FunCoup networks, only pathway genes included in the networks are utilized in the tool. To visualize how many of the unique pathway genes are represented in the FunCoup network, we calculated the fraction of genes in the networks represented as genes in a pathway for each of the pathway databases (see Fig. 4). Here, we could see that mapping the genes for *H.sapiens* retrieved from KEGG on the FunCoup network, covers close to 50% of the genes in the network with a cutoff of 0.8. The number of pathways and unique pathway genes included in PathBIX from each pathway database, for each species, can be found in Supplementary Table S1.

### 3.3 Using PathBIX

To illustrate the usability of PathBIX, we ran the analysis with the gene set CHIN_BREAST_CANCER_COPY_NUMBER_UP for *H.sapiens* against the KEGG database, with a network cutoff of 0.95. This gene set is provided as one of the example gene sets in the tool. The results page of PathBIX when running this analysis can be found in Figure 1. This gene set is obtained from MsigDB (Subramanian *et al.*, 2005) and includes 28 genes that were observed to have copy number aberration gains in more than 15% of 148 primary breast cancer tumor samples (Chin *et al.*, 2007). The gene set originating from the common regions of gains was seen in the study to include oncogenes like ERBB2 as well as tumor suppressor genes, which are common genes not only in breast cancer but in many different cancer types. The PathBIX analysis resulted in 21 pathways having an FWER lower than 0.05. Among the top 10 results, seven pathways were cancer pathways, with ‘Breast cancer’ being placed among the top five at an FWER of 2.35e-3, closely following pathways ‘Prostate cancer’, ‘Pathways in cancer’ and ‘Gastric cancer’, the top-scoring pathway with an FWER of 1.91e-4. Among the top pathways, ‘Thyroid Hormone Signaling Pathway’ is found. Several studies have shown that thyroid hormones can play an important role in cancer cell proliferation, and that hyperthyroidism is correlated to several cancer types, including breast cancer (Liu *et al.*, 2019; Uzair *et al.*, 2019). The occurrence of the ‘Cell cycle’ pathway among the top 10 pathways can be explained by the cell cycle activity often being aberrant in human cancers, and if dysregulated can cause the abnormal cell growth seen in different types of cancer (Otto and Sicinski, 2017).

## 4. Discussion

Here we present PathBIX, a web application for reliable crosstalk-based pathway enrichment analysis. Other websites for network-based pathway enrichment rely on methods that are prone to find false positives (Castresana-Aguirre and Sonnhammer, 2020) because their statistical models assume that pathways can be treated like random gene sets. This applies to EviNET, which is based on NEArender and computes the crosstalk significance by only taking the degrees of the query gene set, the pathway and the network into account. PathwAX uses the BinoX algorithm which computes the crosstalk significance by randomizing the networks and thereby loses some of the properties of the pathway. The main strength of PathBIX resides in providing more reliable results, by using a robust method called ANUBIX which had a much lower false-positive rate than previous methods when benchmarked with random gene sets (Castresana-Aguirre and Sonnhammer, 2020). ANUBIX keeps each pathway’s topology intact and builds its null model by sampling random gene sets of the same size as the query to compute the crosstalk significance to the pathway.

Because different pathway databases use different pathway boundary definitions (Domingo-Fernández *et al.*, 2019), major differences can be obtained in the results from the pathway enrichment analysis depending on the database selected for the analysis. In order to take this into account, and include a wider variety of pathway definitions, PathBIX includes three different pathway databases, KEGG, Reactome and WikiPathways. For certain queries, it can happen that one database yields very few enriched pathways while another one yields dozens. While many pathways overlap considerably between databases, they have somewhat different focuses. For instance, KEGG contains many disease pathways, and Reactome many signaling pathways. This can guide the users to select the pathway database best suiting their needs, as well as give a wider range of annotations by running and comparing the results from different pathway databases.

## Supplementary Material

vbab010_Supplementary_Data

## References

[vbab010-B1] Altenhoff A.M. et al (2020) The Quest for Orthologs benchmark service and consensus calls in 2020. Nucleic Acids Res., 48, W538–W545. 32374845 10.1093/nar/gkaa308 PMC7319555

[vbab010-B3] Carbon S. et al; Web Presence Working Group. (2009) AmiGO: online access to ontology and annotation data. Bioinformatics, 25, 288–289. 19033274 10.1093/bioinformatics/btn615 PMC2639003

[vbab010-B4] Castresana-Aguirre M. , Sonnhammer E.L.L. (2020) Pathway-specific model estimation for improved pathway annotation by network crosstalk. Sci. Rep., 10, 13585. 32788619 10.1038/s41598-020-70239-z PMC7423893

[vbab010-B5] Chin S.-F. et al (2007) Using array-comparative genomic hybridization to define molecular portraits of primary breast cancers. Oncogene, 26, 1959–1970. 17001317 10.1038/sj.onc.1209985

[vbab010-B6] Domingo-Fernández D. et al (2019) ComPath: an ecosystem for exploring, analyzing, and curating mappings across pathway databases. NPJ Syst. Biol. Appl., 5, 10. 30564458 10.1038/s41540-018-0078-8 PMC6292919

[vbab010-B7] Fabregat A. et al (2018) Reactome graph database: efficient access to complex pathway data. PLOS Comput. Biol., 14, e1005968. 29377902 10.1371/journal.pcbi.1005968 PMC5805351

[vbab010-B8] Glaab E. et al (2012) EnrichNet: network-based gene set enrichment analysis. Bioinformatics, 28, i451–i457. 22962466 10.1093/bioinformatics/bts389 PMC3436816

[vbab010-B9] Huang D.W. et al (2009) Systematic and integrative analysis of large gene lists using DAVID bioinformatics resources. Nat. Protoc., 4, 44–57. 19131956 10.1038/nprot.2008.211

[vbab010-B10] Jaiswal P. et al (2002) Gramene: development and integration of trait and gene ontologies for rice. Comp. Funct. Genomics, 3, 132–136. 18628886 10.1002/cfg.156 PMC2447246

[vbab010-B11] Jeggari A. et al (2018) EviNet: a web platform for network enrichment analysis with flexible definition of gene sets. Nucleic Acids Res., 46, W163–W170. 29893885 10.1093/nar/gky485 PMC6030852

[vbab010-B12] Kanehisa M. et al (2021) KEGG: integrating viruses and cellular organisms. Nucleic Acids Res., 49, D545–D551. 33125081 10.1093/nar/gkaa970 PMC7779016

[vbab010-B13] Khatri P. et al (2012) Ten years of pathway analysis: current approaches and outstanding challenges. PLoS Comput. Biol., 8, e1002375. 22383865 10.1371/journal.pcbi.1002375 PMC3285573

[vbab010-B14] Liu,Y.-C. et al (2019) Molecular Functions of Thyroid Hormone Signaling in Regulation of Cancer Progression and Anti-Apoptosis. *International Journal of Molecular Sciences*, 20, 4986. 31600974 10.3390/ijms20204986 PMC6834155

[vbab010-B15] Lock A. et al (2020) Community curation in PomBase: enabling fission yeast experts to provide detailed, standardized, sharable annotation from research publications. Database, 2020, 10.1093/database/baaa028 PMC7192550 32353878

[vbab010-B16] Martens M. et al (2021) WikiPathways: connecting communities. Nucleic Acids Res., 49, D613–D621. 33211851 10.1093/nar/gkaa1024 PMC7779061

[vbab010-B17] Mubeen S. et al (2020) Corrigendum: the impact of pathway database choice on statistical enrichment analysis and predictive modeling. Front. Genet., 11, 436. 32411185 10.3389/fgene.2020.00436 PMC7201094

[vbab010-B18] Nguyen T.-M. et al (2019) Identifying significantly impacted pathways: a comprehensive review and assessment. Genome Biol., 20, 203. 31597578 10.1186/s13059-019-1790-4 PMC6784345

[vbab010-B19] Ogris C. et al (2016) PathwAX: a web server for network crosstalk based pathway annotation. Nucleic Acids Res., 44, W105–W109. 27151197 10.1093/nar/gkw356 PMC4987909

[vbab010-B20] Ogris C. et al (2017) A novel method for crosstalk analysis of biological networks: improving accuracy of pathway annotation. Nucleic Acids Res., 45, e8. 27664219 10.1093/nar/gkw849 PMC5314790

[vbab010-B21] Otto T. , Sicinski P. (2017) Cell cycle proteins as promising targets in cancer therapy. Nat. Rev. Cancer, 17, 93–115. 28127048 10.1038/nrc.2016.138 PMC5345933

[vbab010-B22] Persson E. et al (2021) Funcoup 5: functional association networks in all domains of life, supporting directed links and tissue-specificity. J. Mol. Biol., 433, 166835. 33539890 10.1016/j.jmb.2021.166835

[vbab010-B24] R Core Team. (2017) *R: A Language and Environment for Statistical Computing, R Foundation for Statistical Computing,* Vienna, Austria.

[vbab010-B25] Rodchenkov I. et al (2020) Pathway commons 2019 update: integration, analysis and exploration of pathway data. Nucleic Acids Res., 48, D489–D497. 31647099 10.1093/nar/gkz946 PMC7145667

[vbab010-B26] Schaefer C.F. et al (2009) PID: the pathway interaction database. Nucleic Acids Res., 37, D674–D679. 18832364 10.1093/nar/gkn653 PMC2686461

[vbab010-B1865863] Stonebraker,M., and , Rowe,L.A. (1986) The design of Postgres. *ACM Sigmod Record*, 15, 340–355.

[vbab010-B27] Subramanian A. et al (2005) Gene set enrichment analysis: a knowledge-based approach for interpreting genome-wide expression profiles. Proc. Natl. Acad. Sci. U. S. A., 102, 15545–15550. 16199517 10.1073/pnas.0506580102 PMC1239896

[vbab010-B28] Szklarczyk D. et al (2021) The STRING database in 2021: customizable protein–protein networks, and functional characterization of user-uploaded gene/measurement sets. Nucleic Acids Res., 49, D605–D612. 33237311 10.1093/nar/gkaa1074 PMC7779004

[vbab010-B31] UniProt Consortium. (2021) UniProt: the universal protein knowledgebase in 2021. Nucleic Acids Res., 49, D480–D489. 33237286 10.1093/nar/gkaa1100 PMC7778908

[vbab010-B32] Uzair I.D. et al (2019) Molecular actions of thyroid hormone on breast cancer cell migration and invasion via cortactin/N-WASP. Front. Endocrinol., 10, 139. 10.3389/fendo.2019.00139 PMC6416158 30899247

[vbab010-B95700707] Van Rossum,G., and , Drake,F.L. *Python 3 Reference Manual* , CreateSpace, Scotts Valley, CA, 2009.

