# Supplementary material for: PathBIX—a web server for network-based pathway annotation with adaptive null models
Source: Bioinform Adv. 2021 Jul 1;1(1):vbab010. doi: 10.1093/bioadv/vbab010 (PMC9710673; doi:10.1093/bioadv/vbab010)
Supplement: vbab010_Supplementary_Data [file vbab010_supplementary_data.docx]

**Supplementary Table 1.** Number of pathways and unique pathway genes included in PathBIX for each species from each of the three pathway databases KEGG, Reactome and WikiPathways. Number of links and unique genes from FunCoup 5 used at a threshold of 0.8 for each species.

| **Species** | **KEGG pathways** | **Unique KEGG genes** | **Reactome Pathways** | **Unique Reactome genes** | **Wiki-**  **Pathway pathways** | **Unique Wiki-**  **Pathway genes** | **Genes in FunCoup 5 (cutoff 0.8)** | **Links in FunCoup 5 (cutoff 0.8)** |
| --- | --- | --- | --- | --- | --- | --- | --- | --- |
| H. sapiens | 329 | 7768 | 289 | 7894 | 622 | 7569 | 12890 | 612276 |
| M. musculus | 325 | 8578 | 211 | 6485 | 192 | 4608 | 11887 | 764192 |
| R. norvegicus | 325 | 8380 | 196 | 6531 | 148 | 3309 | 10825 | 856077 |
| C. familiaris | 325 | 6867 | 181 | 5935 | 44 | 1637 | 8476 | 600209 |
| G. gallus | 163 | 4590 | 151 | 3099 | 40 | 1396 | 5819 | 196525 |
| B. taurus | 325 | 7404 | 57 | 2910 | 272 | 4674 | 11897 | 853419 |
| D. rerio | 162 | 7623 | 190 | 5017 | 83 | 2462 | 9467 | 755536 |
| S. scrofa | 325 | 5086 | 124 | 3633 | - | - | 5606 | 248186 |
| C. intestinalis | 119 | 1783 | - | - | - | - | 4260 | 308820 |
| D. melanogaster | 131 | 2451 | 189 | 2862 | 26 | 515 | 6065 | 446105 |
| C. elegans | 129 | 2483 | 220 | 3128 | 60 | 753 | 8172 | 940513 |
| S. cerevisiae | 109 | 2062 | 68 | 1063 | 113 | 862 | 4803 | 302477 |
| A. thaliana | 128 | 4988 | 117 | 3156 | 25 | 634 | 13039 | 1412947 |
| S. pombe | 106 | 1733 | 125 | 1164 | - | - | 2373 | 68958 |
| P. falciparum | 80 | 1049 | 49 | 457 | - | - | 1325 | 40492 |
| O. sativa | 129 | 3911 | 117 | 2160 | 8 | 331 | 6139 | 840379 |
| B. subtilis | 103 | 1233 | - | - | - | - | 2629 | 12145 |
| E. coli | 108 | 3124 | - | - | 6 | 301 | 6162 | 233128 |
| S. solfataricus | 94 | 726 | - | - | - | - | 435 | 6277 |
| M. jannaschii | 74 | 537 | - | - | - | - | 320 | 3532 |
| D. discoideum | 99 | 1692 | 245 | 1878 | - | - | 3321 | 212115 |
